# Biofilm formation and antibiotic resistance of *Klebsiella pneumoniae* isolated from clinical samples in a tertiary care hospital, Klaten, Indonesia

**DOI:** 10.1186/s12919-019-0176-7

**Published:** 2019-12-16

**Authors:** Hera Nirwati, Kian Sinanjung, Fahrina Fahrunissa, Fernando Wijaya, Sarastia Napitupulu, Vania P. Hati, Mohamad S. Hakim, Andreanita Meliala, Abu T. Aman, Titik Nuryastuti

**Affiliations:** 1 Department of Microbiology, Faculty of Medicine, Public Health and Nursing, Universitas Gadjah Mada, Yogyakarta, 55281 Indonesia; 2 Laboratory of Clinical Microbiology, Yogyakarta General Hospital, Yogyakarta, Indonesia; 3 Undergraduate Program of Medicine, Faculty of Medicine, Public Health and Nursing, Universitas Gadjah Mada, Yogyakarta, Indonesia; 4 Department of Physiology, Faculty of Medicine, Public Health and Nursing, Universitas Gadjah Mada, Yogyakarta, Indonesia

**Keywords:** *K. pneumoniae*, Multidrug resistance, Biofilm producer

## Abstract

**Background:**

*Klebsiella pneumoniae (K. pneumoniae)* is a common cause of health-care associated infections (HAIs) and has high levels of antibiotic resistance. These bacteria are well-known for their ability to produce biofilm. The purpose of this study was to identify the antibiotic resistance pattern and biofilm-producing capacity of *K. pneumoniae* isolated from clinical samples in a tertiary care hospital in Klaten, Indonesia.

**Methods:**

*K. pneumoniae* was isolated from inpatients in Soeradji Tirtonegoro Hospital Klaten from June 2017 to May 2018. Identification of *K. pneumoniae* isolate was done by analyzing colony morphology, microscopic examination, and by performing biochemical testing. Testing of antibiotics susceptibility and biofilm-producing capacity used the Kirby-Bauer disk diffusion method and adherence quantitative assays, respectively.

**Results:**

A total of 167 (17.36%) *K. pneumoniae* isolates were isolated from 962 total clinical bacterial isolates during the study. Most of them were collected from patients aged more than 60 years old and were mainly obtained from respiratory specimens (51.50%). Most of *K. pneumoniae* isolates were extensively resistant to antibiotics. A more favorable profile was found only towards meropenem, amikacin, and piperacillin-tazobactam, showing 1.20%; 4.79% and 10.53% of resistance, respectively. The overall proportion of multidrug-resistant *K. pneumoniae* isolates was 54.49%. In addition, 148 (85.63%) isolates were biofilm producers, with 45 (26.95%) isolates as strong, 48 (28.74%) isolates as moderate, and 50 (29.94%) isolates as weak biofilm producers.

**Conclusion:**

Most of the *K. pneumoniae* isolates demonstrated resistance to a wide range of antibiotics and are biofilm producers.

## Background

The emergence of antimicrobial resistance (AMR) is a rapidly increasing challenge in today’s healthcare institutions worldwide. Pathogenic bacteria, for example, *Klebsiella pneumoniae* are quickly developing multidrug resistant (MDR) strains and commonly pose a serious threat to the patients because of an increased fatality rate due to the reduced effectiveness of therapy options. *K. pneumoniae* is known to be responsible for community acquired infections although recently it is routinely observed as a major cause of hospital acquired pathogens. *K. pneumoniae* has been observed to develop resistance to antibiotics more easily than most bacteria through the production of enzymes such as Extended Spectrum β-Lactamase (ESBLs) and Carbapenemase [1–3]. The most important risk factor of AMR is antibiotic exposure**.** Some of the main contributors in the emergence and spread of highly resistant bacteria for health-care associated infections (HAIs) are the intensive and prolonged use of antibiotics in the hospital setting [4].

*K. pneumoniae* also plays an important role in spreading antimicrobial resistance genes from bacteria in the environment to clinically important bacteria [5]. There are multiple mechanisms of antimicrobial resistance which will negatively affect the therapeutic outcomes. The World Health Organization (WHO) declared antibiotic resistance as one of the three major problems in the world [3].

*K. pneumoniae* is a Gram-negative encapsulated bacteria that thrives in the mucosal surfaces of mammals and in the soil, vegetation, and water. In humans, *K. pneumoniae* typically will colonize the oropharynx and gastrointestinal tract, from where it can easily enter the circulation and other tissues causing infections such as bacteremia, septicemia, surgical site infection, urinary tract infection, hospital acquired pneumonia, and ventilator-associated pneumonia. It also contributes to the high frequency of opportunistic infections that occur among patients with immunocompromised situations, such as bladder neuropathy or diabetes mellitus [6, 7].

*K. pneumoniae* is also known for its capability to form biofilms, which are communities of bacteria embedded in an extracellular matrix. This matrix consists of proteins, exopolysaccharides, DNA, and lipopeptides [8]. In 1988, LeChevallier et al. first described *K. pneumoniae* and the biofilm-forming phenomenon [9]. *K. pneumoniae* has some virulence factors such as capsule polysaccharide, lipopolysaccharide, type 1 and type 3 fimbriae, outer membrane proteins and determinants for iron acquisition and nitrogen source usage. *K. pneumoniae* used these virulence factors for survival and for evade from immune system during infection as well as biofilm formation itself [10, 11]. *K. pneumoniae* can produce a thick layer of extracellular biofilm that supports the bacterial attachment to living or non-living surfaces, protecting antibiotics penetration and reducing its effects [12].

In 2017, there were 213 (19.28%) *K. pneumoniae* isolates from 1105 bacteria isolated in Soeradji Tirtonegoro hospital in Klaten, Indonesia. In 2005, a study conducted in a hospital in Surabaya, Indonesia reported the prevalence of ESBLs was 28% among clinical isolates of *K. pneumoniae* [13]*.* These antimicrobial-resistant bacteria have become a worldwide problem and there is still very limited data regarding biofilm producing capacity and antimicrobial resistence of *K. pneumoniae* in Indonesia. Biofilm forming capacity is demonstrated to have a greater resistance to antibiotics. Further studies are needed to describe the pattern of antibiotic resistance and the capacity to produce biofilm of *K. pneumoniae* clinical isolates in Indonesia. Thus, the purpose of this study was to identify the antibiotic resistance patterns, and the biofilm-producing capacity of *K. pneumoniae* clinical isolates in the Soeradji Tirtonegoro hospital in Klaten, Indonesia.

## Methods

### Subjects

Using a cross-sectional design, this observational research studied *K. pneumoniae* clinical isolates from inpatients in Soeradji Tirtonegoro Hospital, a tertiary care hospital Klaten, Indonesia, during June 2017 – May 2018.

### Isolation, identification and antibiotic susceptibility tests of *K. pneumoniae*

Identification of *K. pneumoniae* isolate was conducted by culturing on McConkey agar, Gram staining and biochemical testing using Microbact™ GNB 24E (Oxoid, UK). Kirby Bauer method was used to perform antibiotic susceptibility tests. The Clinical and Laboratory Standards Institute 2015 was used to classify as sensitive, intermediate or resistant bacteria [14]. *K. pneumoniae* isolates that showed résistance to three or more different classes of antimicrobials were classified as multidrug-resistant (MDR) *K. pneumoniae* [15].

### Biofilm formation assay

A quantitative adherence assay was employed to perform biofilm formation assay [16]. An overnight culture at 37 °C in trypticase soy broth (TSB) was conducted for each isolate. Subsequently, 2 μL of cell suspension was inoculated in sterile 96 well-flat bottom polystyrene microtitre plates contained 198 μl of TSB. Negative control wells that contained 200 ul of un-inoculated TSB also included in each test. Incubation was done at 37 °C for 24 h. With 200 μL phosphate-buffered saline (PBS), the wells were gently washed 3 times. The wells were dried in an inverted position. Biofilm mass was stained with 50 μL of 0.1% crystal violet. With 200 μL of distilled water, the wells were gently washed 3 times and dried in an inverted position. Finally, the wells were dissolved in 200 μL of 5% isopropanol acid to solubilize the stained biofilm mass. Optical density (OD) measurement was done by using a microplate reader at 570 nm. Each isolate or negative control was tested for 8–12 wells, and the mean OD was determined. Optical density cut-off (ODc) was assigned as an average OD of negative controls + (3× standard deviation (SD) of negative controls). Isolate with OD ≤ ODc categorized as non-biofilm producer. Meanwhile, the isolate was categorized as biofilm producer consisting of weak biofilm producer if 2 × ODc < OD ≤4 × ODc; moderate biofilm 2 × ODc < OD ≤4 × ODc; and strong biofilm producer if OD > 4 × ODc [16].

### Statistical analysis

Frequencies and percentages were used to describe the variables in this study. Associations between the antibiotic sensitivity pattern and the biofilm producing capacity of *K. pneumoniae* were tested by Chi-square tests using STATA 13 software (STATA, College Station, Texas). The results were presented as prevalence ratios with a 95% confidence interval. Statistical significance was set if *p*-value < 0.05.

### Ethical approval

The Medical and Health Research Ethics Committee, Faculty of Medicine, Public Health and Nursing, Universitas Gadjah Mada, Yogyakarta, Indonesia permitted this study with number: KE/FK/0862/EC/2017.

## Results

### Characteristics of clinical samples

From June 2017 to May 2018, 167 (17.36%) *K. pneumoniae* isolates were examined from 962 total clinical bacterial isolates at Soeradji Tirtonegoro Hospital, Klaten, Indonesia. *K. pneumoniae* isolates were isolated from 107 male (64.07%) and 60 female (35.93%) patients. Most of *K. pneumoniae* were isolated from patients aged more than 60 years old (38.10%) (Table 1). *K. pneumoniae* samples were mostly isolated from respiratory specimens (51.50%) and pus (16.17%) (Table 1).

**Table 1:** Characteristics of patients from which *K. pneumoniae* were isolated

Characteristics		Total	
		n	%
Sex	Male	107	64.07
	Female	60	35.93
Age (years)	< 15	50	29.76
	15–59	53	31.55
	≥ 60	64	38.10
Sampling Location	Non- ICU	119	71.26
	ICU/ PICU/ICCU	48	28.74
Clinical Specimens	Respiratory specimens (sputum, bronchial washing, and tracheal aspiration)	86	51.50
	Pus	27	16.17
	Blood	18	10.78
	Urine	12	7.19
	Stool	6	3.59
	Wound swab	5	2.99
	Peritoneal fluid	5	2.99
	Tissue	2	1.20
	Others	6	3.59
Total		167	100

*ICU* Intensive care unit, *PICU* Pediatric intensive care unit

*ICCU* Intensive cardiac care unit

### Antibiotic susceptibility pattern

Most of *K. pneumoniae* were resistant to a wide range of antibiotics. Among biofilm producer isolates, *K. pneumoniae* had only a good sensitivity to meropenem (98.60%), amikacin (95.80%), and piperacillin-tazobactam (90.00%). In contrast, among non-biofilm producer isolates, *K. pneumoniae* showed good sensitivity to meropenem, levofloxacin, amikacin, piperacillin-tazobactam and ciprofloxacin with 100.00%; 95.83%; 91.67%; 87.50%, and 86.67% of sensitivity respectively (Table 2).

**Table 2:** Antibiotic susceptibility pattern and biofilm producing capacity of *K. pneumoniae* clinical isolates

			Biofilm Producer				Non-Biofilm Producer			
No	Antibiotics	Number tested	Sensitive		Resistant		Sensitive		Resistant	
			n	%	n	%	n	%	n	%
1	Ampicillin	167	3	2.10	140	97.90	1	4.17	23	95.38
2	Cefazolin	139	42	34.71	79	65.29	8	44.44	10	55.56
3	Gentamicin	167	88	61.54	55	38.46	17	70.83	7	29.17
4	Tobramycin	167	77	53.85	66	46.15	18	75.00	6	25.00
5	Amikacin	167	137	95.80	6	4.20	22	91.67	2	8.33
6	Amoxicillin-Clavulanic acid	139	75	61.98	46	38.02	13	72.22	5	27.78
7	Piperacillin-Tazobactam	38	27	90.00	3	10.00	7	87.50	1	12.50
8	Cefuroxime	167	59	41.26	84	58.74	14	58.33	10	41.67
9	Cefepime	166	62	43.66	80	56.34	15	62.50	9	37.50
10	Ceftriaxone	167	62	43.36	81	56,64	15	62.50	9	37.50
11	Ciprofloxacin	80	36	55.38	29	44.62	13	86.67	2	13.33
12	Levofloxacin	166	109	76.76	33	23.24	23	95.83	1	4.17
13	Meropenem	167	141	98.60	2	1,40	24	100.00	0	0.00
14	Trimethoprim-Sulfamethoxazole	146	60	46.88	68	53,13	12	66.67	6	33.33

### Biofilm formation detection

In this study, among the 167 *K. pneumoniae* isolates tested, there were 143 (85.63%) isolates as biofilm producer and 24 (14.37%) isolates that were not biofilm producers. Among biofilm producers, there were 45 (26.95%) isolates as strong, 48 (28.74%) isolates as moderate, and 50 (29.94%) isolates identified as weak biofilm producers (Table 3). *K. pneumoniae* MDR isolates were found in 91 (54.5%) isolates and 76 (45.5%) isolates were non-MDR (Table 4). There was no significant association between *K. pneumoniae* MDR and biofilm production capacity based on the statistical analysis using chi-square tests (Table 4).

**Table 3:** Biofilm producing capacity of *K. pneumoniae* clinical isolates

Characteristics	Number (%)
Nonbiofilm producer	24 (14.37)
Weak biofilm producer	50 (29.94)
Moderate biofilm producer	48 (28.74)
Strong biofilm producer	45 (26.95)
Total	**167 (100%)**

**Table 4:** Association between *K. pneumoniae* multidrug-resistant (MDR) and biofilm producing capacity

		Biofilm producer		*p*-value	PR^a^	CI 95%
		positive	negative			
Multidrug resistant (MDR)	positive	82 (49.10%)	9 (5.39%)	0.07	1.12	0.9852–1.279
	negative	61 (36.53%)	15 (8.98%)			
Total		143	24			

*CI* Confidence interval; ^a^ *PR* Prevalence ratio

## Discussion

In this study, the proportion of *K. pneumoniae* isolates was 17.36% of the total clinical bacterial isolates during June 2017–May 2018. This proportion should raise serious concerns because *K. pneumoniae* is one of the most important causes of MDR infections in the world. These bacteria are often associated with HAIs and highly contagious outbreaks in hospitals with increased mortality rate and longer stays in the hospitals, all of which results in inflated healthcare costs [17].

Most of the *K. pneumoniae* isolates in this study were collected from male patients. This result was in line with Osagie et al. [18] who collected the samples from 5 primary health care centers in Nigeria and reported that *K. pneumoniae* infection was higher in males than females. Akter et al. [19] also reported that male patients had a higher risk to get Klebsiella infection than females. The association between sex and the incident of *K. pneumoniae* was associated with poor lifestyle choices in the form of smoking and alcoholism [18]. However, no statistically significant differences between female and male subjects were reported in those studies.

Most of *K. pneumoniae* in this study were obtained from patients aged 18 to 65 years of age. This finding differs from a previous study that revealed most *K. pneumoniae* isolates were isolated from patients over 70 years old [20]. Meanwhile, another recent study suggested that a greater number of *K. pneumoniae* isolates were obtained from patients aged between 40 to 65 years old [21]. The differences in terms of patient’s age distribution could be related to the strength of the immune system response, which is expected to decline in senescence. Patients aged under 40 years tend to have stronger immune systems, thus giving more pressure to *K. pneumoniae* to fight the immunity of the host [22]. On the contrary, an increased age leads to a higher risk of *K. pneumoniae* infection because of increased incident of comorbid illness [23].

*K. pneumoniae* isolates were mainly isolated from respiratory specimens. Ashurst and Dawson [7] emphasized that *K. pneumoniae* typically colonizes human mucosal surfaces of the oropharynx and gastrointestinal tract. For this reason, *K. pneumoniae* is considered to be the most common cause of hospital-acquired pneumonia in the United States. This finding is similar to a study by Wang et al. [24] which reported that the respiratory tract was the main infection site of *K. pneumoniae* in the Republic of China. In comparison, Seifi et al. [6] who collected samples from 2 hospitals in Tehran reported that *K. pneumoniae* samples were isolated from urine, surgical wounds, sputum and blood with the percentage of 61.7, 18.1, 11.7, and 8.5% respectively.

Most of *K. pneumoniae* was resistant to various antibiotics, with ampicillin, cefazolin, and cefuroxime being the least effective for *K. pneumoniae* while amikacin, piperacillin-tazobactam, and meropenem had the most favorable profile. This report is supported by the study conducted by Madahiah [25] that found *K. pneumoniae* isolates were 100% resistant to ampicillin and 100% sensitive to amikacin. Ciprofloxacin and amoxicillin-clavulanic acid showed 38.75% and 36.69% resistance, respectively. This finding is similar to that of Cepas et al. [26] that reported 40% of *K. pneumoniae* strains were resistant to ciprofloxacin and amoxicillin-clavulanic acid.

Antibiotic exposure is the most crucial factor of antimicrobial resistance**.** The growth of antibiotic resistance are involving many factors such as the use of antibiotics in the hospital, in community, even in animal production, agriculture, and environment. As a consequence of the capability in purchasing antibiotics freely without prescription, therefore, antibiotics are used excessively. In the health service setting, intensive and prolonged use of antibiotics are very likely the main underlying factor in the widespread transmission of difficult-to-cure antibiotic-resistant nosocomial infections [4].

The overall proportion of MDR *K. pneumoniae* isolates in this study was 54.49%. This high percentage of MDR *K. pneumoniae* is supported by some previous studies. Cepas et al. [26] informed that 38% of *K. pneumoniae* strains were MDR. Manjula et al. [27] showed that 37 (90.2%) of 41 isolates were MDR. It was also found that the majority of the MDR *K. pneumoniae* isolates showed high resistance towards penicillin, cephalosporin, fluoroquinolone, aminoglycoside, and sulfonamide. This result is in line with a previous study by Moini et al. [28] which reported that 46.6% of the *K. pneumoniae* isolates were MDR and the high resistance was shown towards ampicillin, third-generation cephalosporins, and aminoglycosides. This MDR pattern exhibited by microbes is causing a great challenge in managing infections, and consequently, it is very important to monitor and optimize antibiotic use through antibiotic stewardship programs. Several studies have shown that treatment with a combination of antibiotics can help to prevent the emergence of new resistant strains, as therapy failures are commonly found in individuals who only receive single antibiotic therapy. It is also important for clinicians and microbiologists to collaborate to warrant effective management of infections as promoted in the Rational Use of Medicine (RUM) Program [29].

In this study, 148 (85.63%) of 167 isolates were biofilm producers. A similar study reported by Hassan et al. [16] stated that out of 110 *K. pneumoniae* tested, 70 isolates (64.7%) were identified as high or moderate biofilm producers and 40 isolates (35.3%) were identified as weak biofilm producers. In another study, Cepas et al. [26] reported that 37.6% of *K. pneumoniae* strains were biofilm producers. Yang and Zhang [20] reported that 62.5% of *K. pneumoniae* isolated from urine, sputum, wound swabs and blood were biofilm producers. Seifi et al. [6] reported that the majority of *K. pneumoniae* (93.6%) were biofilm producer and only 6.4% were not biofilm producers. Among biofilm producer strains, 33% were categorized as strong biofilm producer, 52.1% as moderately biofilm producers and 8.5% as weak biofilm producers. The capacity to form biofilms was different from each isolate because in general several factors are influencing the capacity such as the physicochemical characteristic of *K. pneumoniae,* physical interaction between the constituents, the type of surface where the biofilm attaches, temperature, pH, etc. [30]. Vuotto et al. [31] highlighted extensively drug resistant (XDR) *K. pneumoniae* biofilm-producing ability correlate to antibiotic resistance profile.

Cepas et al. [26] looked for possible relation of antimicrobial resistance and the ability to form biofilm between the collected samples of *E. coli, K. pneumoniae*, and *P. aeruginosa*. There was no statistically significant relationship between general MDR and biofilm formation in three Gram-negative species because the MDR isolates did not show any greater disposition to becoming a stronger biofilm producer compared to non-MDR isolates. However, they reported some correlations between biofilm formation and resistance to specific antibiotics. Resistance to gentamicin and ceftazidime was correlated with biofilm formation in *E. coli*, as well as ciprofloxacin in *P. aeruginosa* and piperacillin-tazobactam and colistin in *K. pneumoniae*. This pattern of resistance should raise grave concerns because colistin is now considered to be the last line treatment choice for *K pneumoniae* [17].

This study showed that antibiotic resistance was greater among *K. pneumoniae* which is biofilm producer than non-biofilm producer. This finding has been reported in many studies. A study by Saha et al. [32] demonstrated that all the biofilm-producing isolates presented more resistant patterns in comparison to non-biofilm producers, however, despite that finding, the protective mechanisms in biofilms are different from those responsible for conventional antibiotics resistance. In biofilms, the protective covering of the adhesive biomaterial that leads to poor antibiotics penetration, the adaptive responses to stress, and the formation of persister cells are hypothesized to constitute a multilayered defense, thus increasing the difficulty in eradication especially when combined with the resistant nature of the bacteria itself [33, 34]. Another study by Alcantar-Curiel et al. [35] proposed that while it appears that antibiotics resistance and bacterial capability to form biofilm play an important role in the global spread of *K. pneumoniae*, the clear relationship between these factors has not been fully identified and elaborated. This finding is confirmed by de Campos et al. [36] explaining that the capacity to form biofilms was not clearly correlated with clonal types of MDR bacteria.

It has been confirmed by several studies that in the majority of cases, a single antibiotic therapy is inadequate to annihilate biofilm-forming infections. In consequence, managing infections with currently accessible antibiotics and evaluating the outcomes have become important and urgent protocols for the successful treatment of biofilm-associated infections [29]. Several studies recommend a combination of antibiotic therapy with macrolides such as erythromycin, clarithromycin, and azithromycin as the main antibiotics for biofilm associated infection due to Gram-negative bacteria because of their high antibiofilm activity inside and outside of living organisms [37]. Taking into account the currently known environmental and bioecological aspects, Wu et al. [29] suggested that apart from the administration of combined antibiotics, removal of infected foreign bodies and source of infection as well as the administration of bacterial quorum sensing inhibitors or biofilm dispersal agents would result in a more effective management for biofilm infections.

Our study showed the antibiotic-resistant bacteria problems in hospital setting which are also reported by some studies [38, 39]. This situation is alarming when considering the quality and quantity of antibiotics prescriptions in most hospitals. Hospital surveillance in Surabaya conducted in 2012 demonstrated that 30.6% of antibiotics were administered without indications supported by susceptibility tests [40]. Thus, the prescription of antibiotics continues to become a formidable challenge in the world, including Indonesia. In the Netherlands, where antibiotic usage and antibiotic resistant bacteria are low, the antibiotics prescription protocols are not optimal as reported by van der Meer [41]. They reported that 15% of antibiotic therapy in surgical and internal medicine wards was not adequate [41].

Resistance mediated by ESBLs consists of penicillins, cephalosporins (including third-generation cephalosporins) and aztreonam. Dumaru et al. [42] performed a study to identify biofilm formation by Gram-negative bacteria and figured out their antibiograms along with the detection of ESBLs and Metallo-beta-lactamases (MBLs) production. There was a statistically significant association between MBL and biofilm production. However, the association between ESBL and biofilm production was not significant [42].

The major cause of inappropriate antibiotic prescribing is due to a lack of education about infection and antibiotic usage. One of the most relevant steps in antibiotic prescribing is an adjustment of initial antibiotic therapy based on the clinical microbiology result [43]. Therefore, it is essential to perform antibiotic susceptibility testing. Collecting clinical samples before antibiotic administration is also a critical point. Many physicians who prescribe antibiotics do not completely understand if their inappropriate prescriptions can have an impact on bacterial resistance development. Adjusting the initial antimicrobial therapy based on the clinical microbiology result will diminish the selection pressure to the microorganism in hospital-based infections. Thus, it is of paramount importance for each hospital to have an antibiotic guidance or stewardship program for all pharmacists and the physicians based on the most accurate microbiological data. In conjunction with this guidance, a continuous effort in hospital surveillance, infection control, and clinical audits must be conducted to fight against the rapid development of antibiotic-resistant pathogens [43].

## Conclusions

Most of the *K. pneumoniae* isolates showed resistance to a wide range of antibiotics. Most of these were also shown to be biofilm producers of various capacities. Global efforts should be intensified to prevent the spread of multi-drug resistant bacteria and eliminate the hospital-born microbes that are causing a dramatic rise in mortality.

## Data Availability

All data analyzed or generated during this study are included in the submission. The raw data are available from the corresponding author on reasonable request.

## Abbreviations

AMR: Antimicrobial resistance
ESBLs: Extended Spectrum β-Lactamases
MBLs: Metallo-beta-lactamases
MDR: Multi-drug resistant
OD: Optical density
ODc: Optical density cut-off
